# A data-driven design for sound absorption of acoustic metamaterials based on large language models

**DOI:** 10.1038/s41598-025-29930-2

**Published:** 2025-12-04

**Authors:** Yongfeng Jiang, Siyang Cao, Han Meng, Runze Zhou, Jianwei Ren, Xiangchao Feng, Cheng Shen, Tianjian Lu

**Affiliations:** 1 State Key Laboratory for Mechanics and Control for Aerospace Structures, Nanjing University of Aeronautics and Astronautics, Nanjing, 210016 People’s Republic of China; 2 MIIT Key Laboratory of Multifunctional Lightweight Materials and Structures, Nanjing University of Aeronautics and Astronautics, Nanjing, 210016 People’s Republic of China; 3 PLA Rocket Force University of Engineering, Xi’an, 710025 People’s Republic of China; 4 Academy of Aerospace Science and Technology Innovation, Beijing, 100088 People’s Republic of China

**Keywords:** Large language model, Machine learning, Data-driven design, Sound absorption, Acoustic metamaterials, Engineering, Materials science, Mathematics and computing

## Abstract

Machine learning (ML)-based data-driven approaches are extensively employed in forward and inverse acoustic metamaterial design, as evidenced by numerous research papers published in recent years. These studies require advanced ML knowledge and coding skills. Furthermore, the proposed ML models lack generalizability, being tailored to specific structures and hard to apply broadly, limiting practical applications. To address these issues, this study establishes two data-driven design strategies—agent interaction and large language model (LLM) fine-tuning—based on LLMs, eliminating the need for specialized ML knowledge. This approach provides a universal user-friendly strategy for acoustic metamaterial design. The agent interaction strategy enables ChatGPT to act as an independent agent, mapping structural parameters to sound absorption coefficients through simple text interactions, thereby facilitating both forward and inverse design. The LLM fine-tuning strategy involves retraining DeepSeek using acoustic metamaterial datasets, adjusting specific model parameters to enable performance prediction or inverse design. Results indicate that the agent interaction strategy can design acoustic metamaterials within one minute solely through dialogue and instruction. The fine-tuned LLM strategy yields design outcomes with higher accuracy compared to the conventional ML model. Additionally, the fine-tuned LLM can evolve into a specialized LLM for the metamaterial domain through continuous fine-tuning. The proposed strategies validate the application potential of LLMs in data-driven metamaterial design and provide significant guidance for advancing this field.

## Introduction

Benefiting from the significant advances in machine learning (ML), ML-based data-driven methods allow structural design to be guided by existing data, rather than relying on the experience, intuition, and assumptions of designers^1–4^. ML-based data-driven design has demonstrated unique advantages such as high reliability, rapid response, and low cost, which have driven further development in numerous scientific fields such as mechanics, mathematics, biology, and chemistry^5–7^. Moreover, ML-based data-driven design is highly effective in solving high-dimensional and inverse problems, simultaneously uncovering the hidden patterns behind them^8–10^. Therefore, a great deal of ML method has been proposed for data-driven design of engineering structures, focusing on purposes of forward design (i.e. fast performance prediction with given structural features), inverse design (i.e. location of structures with desired performance) or optimization. For instance, the Convolutional Neural Network (CNN) excels at extracting hidden information about structural performance from configurations, and the Long Short-Term Memory neural network (LSTM) is capable of handling prediction tasks with temporal features^11–13^. Additionally, generative ML models, such as Generative Adversarial Network (GAN) and Variational Autoencoders (VAE), can offer unconventional and creative design solutions in a short period^14–16^. In short, ML-based data-driven steers the design of engineering structures toward a direction that is free from trial and error, independent of experience, and focused on efficiency and creativity.

However, despite the aforementioned advantages, traditional ML-based data-driven methods pose significant barriers for application in structural design due to their demanding expertise requirements. On one hand, the rapid evolution of the ML field has led to an increasing number of models adept at various design tasks, with deeper network architectures to handle complex, high-dimensional data^17,18^. This necessitates designers to possess a robust grasp of ML theory to select appropriate models and architectures for specific tasks. On the other hand, traditional ML-based methods demand designers to have strong coding abilities to facilitate the entire design process. Despite the availability of code assistance tools, the traditional ML-based methods still present a challenge for designers without coding experience. As a result, traditional ML-based methods are accessible primarily to designers with specialized knowledge and skills, thereby limiting the broader applicability. Moreover, as these models are typically problem-specific, a trained ML model is limited to tasks related to its training dataset. The constrained data processing capabilities hinder the evolution of these models into domain-specific tools through data augmentation and knowledge integration, limiting their generalizability when applied to other problems within the field.

Fortunately, the emergence of large language models (LLMs) has fundamentally reshaped perceptions of artificial intelligence (AI)^19–21^. As ML models trained on massive datasets, LLMs delve into various aspects of daily life and professional activities across multiple domains, such as medicine, finance, and other scientific fields^22–24^. More importantly, LLMs are positioned to challenge the conventional ML-based data-driven design paradigm with the robust capabilities, user-friendly interfaces, high adaptability, and diverse interaction methods^25–29^.

Currently, LLMs are predominantly employed as tools for coding assistance, data analysis, information search and writing in the majority of research and practical applications. However, with the powerful data analysis and interaction capabilities, LLMs are expected to further generalize data-driven design by lowering its entry threshold. This would enable tasks such as data processing, model training, and performance prediction to be executed through conversational interfaces, eliminating the need for designers to possess coding skills or in-depth knowledge of ML theory. At the same time, as models featuring billions of network structure parameters, LLMs have been proved to possess the capacity to assimilate domain-specific knowledge through fine-tuning, thereby enhancing their problem-solving abilities within specific fields and transitioning from general-purpose LLMs to domain-specific ones^30–35^. More recently, within the last one or two years, there has been a growing interest in leveraging LLMs in structural design. Naghavi et al^36^. realized the design, reconstruction, and generation of complex configurations of porous metamaterials by integrating LLMs with a conventional variational autoencoder. Their results demonstrated that the LLM possesses a remarkable capability in reconstructing local features of porous structure. Lu et al^37^. accurately predicted the absorptive spectra of dielectric metamaterials through LLM fine-tuning strategy, achieving predictive performance comparable to that of conventional ML models. Kim et al^38^. applied LLM to the design of nanophotonic device and found that LLM has the potential to learn knowledge through context. Their research demonstrates that LLM can accelerate structural design and reduce design barriers. It is noteworthy that while LLMs have begun to be applied in the field of structural design, the current research is still in its nascent stage, with only isolated case studies. To date, there is a lack of research on the applicability of LLMs to acoustic metamaterials characterized by high-dimensional parameters, complex multi-peak sound absorption curves, and ill-posed inverse problems. Furthermore, although previous studies have predominantly employed fine-tuning strategies, the feasibility of agent interaction strategy has not been thoroughly validated.

Gaining insight from above discussion, a data-driven design for acoustic metamaterials (AMMs) based on LLMs is conducted in this study. AMMs are artificial structures or materials that exhibit unparalleled research and application significance in the engineering domain, owing to their exceptional acoustic and multifunctional performance and lightweight characteristics^39–41^. The design of AAMs have garnered significant interest over the past decade. Dong et al^42^. Offered a comprehensive overview of latest advances of AMM. They systematically reviewed the inverse design approaches of AMMs and introduced for the first time the phononic structures genome engineering as an extension of inverse design in their paper. As highlighted by Dong and other researchers, the performance of AMMs depends intrinsically on their structural configurations, data-driven design has been considered an efficient and cost-effective strategy for the design of AMMs^42–47^. Considering the different needs of designers, two strategies are adopted within the LLMs-based data-driven design framework: agent interaction and LLM fine-tuning. The agent interaction strategy aims to dismantle the professional barriers associated with ML in data-driven design, enabling tasks such as predicting the sound absorption performance and inverse design of structural parameters only through dialogue. Considering accuracy and knowledge integration, a LLM fine-tuning strategy is also developed by training LLMs using existing AMMs datasets. Additionally, the LLM fine-tuning strategy is expected to evolve generic LLMs into AMM-specific LLMs through subsequent data augmentation and knowledge integration.

This paper is structured as follows: Sect. “Methods” outlines the methodology for the agent interaction and LLM fine-tuning strategies. Section “Results and discussion” discusses the results of the above strategies in the forward prediction and inverse design of sound absorption performance of AMMs. A comprehensive evaluation of the above strategies is also conducted in Sect. “Results and discussion”. Finally, conclusions are provided in Sect. “Conclusions”.

## Methods

### Dataset

**Fig. 1:**
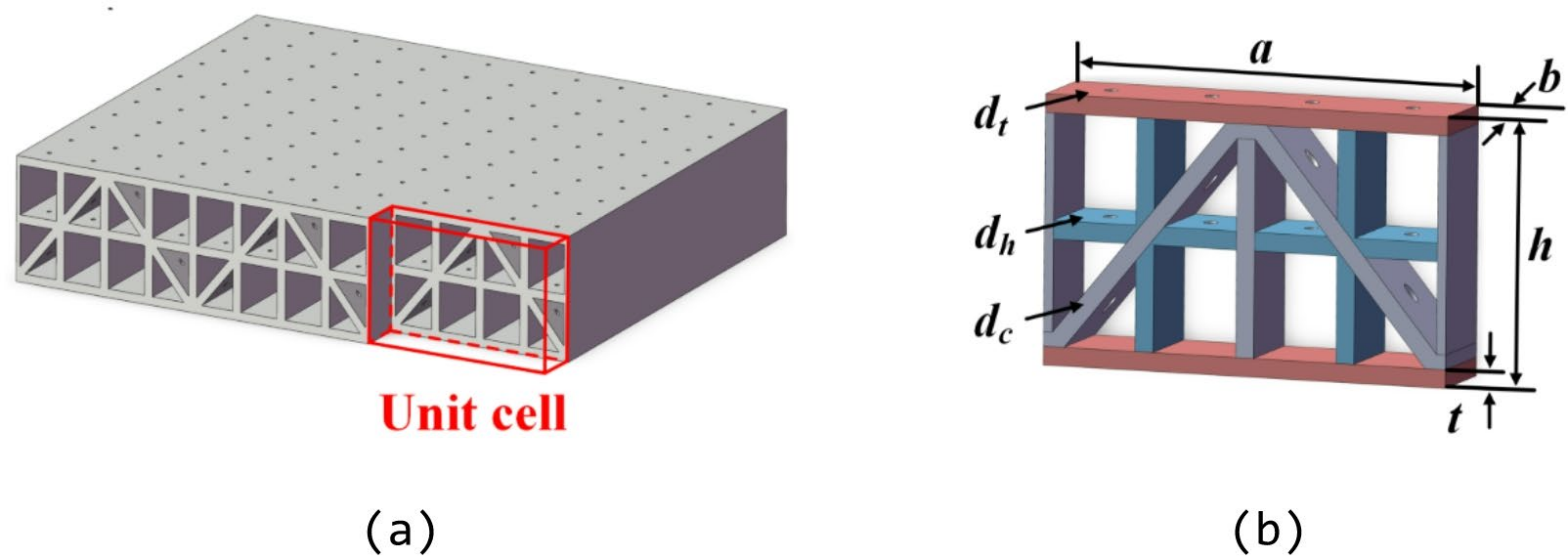
The schematic diagram of (**a**) the micro-perforated N–H cored sandwich metamaterial and (**b**) the unit cell.

The micro-perforated N–H cored sandwich metamaterial, as illustrated in Fig. 1a, is selected as a case study to explore the efficacy of LLMs in data-driven design for AMMs^48^. As shown in Fig. 1b, the dimensions of the unit cell are specified as length *a*, width *b*, and height *h*. The diameter of the micro-perforations on the facesheet, horizontal insertion and corrugation are defined as ${d}_{f}$, ${d}_{h}$ and ${d}_{c}$, respectively. Additionally, the thickness of all panels within the unit cell is represented by *t*. As shown in Fig. 1b, the unit cell can be divided into four subunits from left to right, with each subunit consisting of three Helmholtz resonators connected in series from top to bottom. Taking one subunit as an example, the surface impedance of its lowest resonant cavity ${Z}_{1l}$ can be determined by the following Eq. ^48^,

1 $$\begin{array}{*{20}c} {Z_{1l} = r_{l} + j\omega m_{l} + \frac{1}{{j\omega C_{l} }}} \\ \end{array}$$

Using the electroacoustic analogy method, the surface impedance of the intermediate resonant cavity $Z_{1m}$ can be determined by

2 $$\begin{array}{*{20}c} {Z_{1m} = r_{m} + j\omega m_{m} + \frac{1}{{j\omega C_{m} }} \times \frac{{Z_{1l} }}{{\frac{1}{{j\omega C_{m} }} + Z_{1l} }}} \\ \end{array}$$

Similarly, the surface impedance of the top resonant cavity $Z_{1t}$ can be expressed as

3 $$\begin{array}{*{20}c} {Z_{1t} = r_{t} + j\omega m_{t} + \frac{1}{{j\omega C_{t} }} \times \frac{{Z_{1m} }}{{\frac{1}{{j\omega C_{t} }} + Z_{1m} }}} \\ \end{array}$$

where ${\text{j}}$ represents the imaginary unit, $\omega$ represents the angular frequency, $r_{{\left( {l, m, t} \right)}}$ and $m_{{\left( {l, m, t} \right)}}$ are defined as the sound resistance and sound mass of the resonant cavity, as

4 $$\begin{array}{*{20}c} {r_{{\left( {l, m, t} \right)}} = \frac{32\mu t}{{p_{{\left( {l, m, t} \right)}} d_{{\left( {c,h,t} \right)}}^{2} }}\left( {\sqrt {1 + \frac{{k_{{\left( {l,m,t} \right)}}^{2} }}{32}} + \frac{{\sqrt 2 k_{{\left( {l,m,t} \right)}} d_{{\left( {c,h,t} \right)}} }}{8t}} \right)} \\ \end{array}$$

5 $$\begin{array}{*{20}c} {m_{{\left( {l, m, t} \right)}} = \frac{t}{{p_{{\left( {l, m, t} \right)}} c}}\left( {1 + \sqrt {\frac{2}{{k_{{\left( {l,m,t} \right)}}^{2} + 18}}} + \frac{{0.85d_{{\left( {c,h,t} \right)}} }}{t}} \right)} \\ \end{array}$$

where ${p}_{(l,m,t)}$ is the porosity of the facesheet of the resonant cavity, ${k}_{\left(l,m,t\right)}={d}_{\left(c,h,t\right)}\sqrt{\omega \rho /4\mu }$, $\rho$ = 1.2 kg/m^3^ and $\mu$ = 1.85 × 10^–5^ Pa/s^−1^ are the air density and viscosity coefficient, $c$ = 343 m/s is the sound velocity under standard conditions. ${C}_{\left(l, m, t\right)}={V}_{\left(l, m, t\right)}/\rho {c}^{2}$ is defined as the acoustic compliance of the resonant cavity, ${V}_{\left(l, m, t\right)}$ is the cavity volume.

Similarly, the surface impedance of the other three subunits ${Z}_{2t}$, ${Z}_{3t}$ and ${Z}_{4t}$ can be determined using the above equation, while the total surface impedance of the unit cell $Z$ can be obtained by connecting the impedances of the four subunits in parallel, which is given as

6 $$\begin{array}{*{20}c} {Z = \frac{4}{{\frac{1}{{Z_{1t} }} + \frac{1}{{Z_{2t} }} + \frac{1}{{Z_{3t} }} + \frac{1}{{Z_{4t} }}}}} \\ \end{array}$$

The sound absorption performance of the AMM is evaluated using the sound absorption coefficient (SAC), defined as

7 $$\begin{array}{*{20}c} {\alpha = 1 - \left| {\frac{Z - \rho c}{{Z + \rho c}}} \right|^{2} } \\ \end{array}$$

**Fig. 2:**
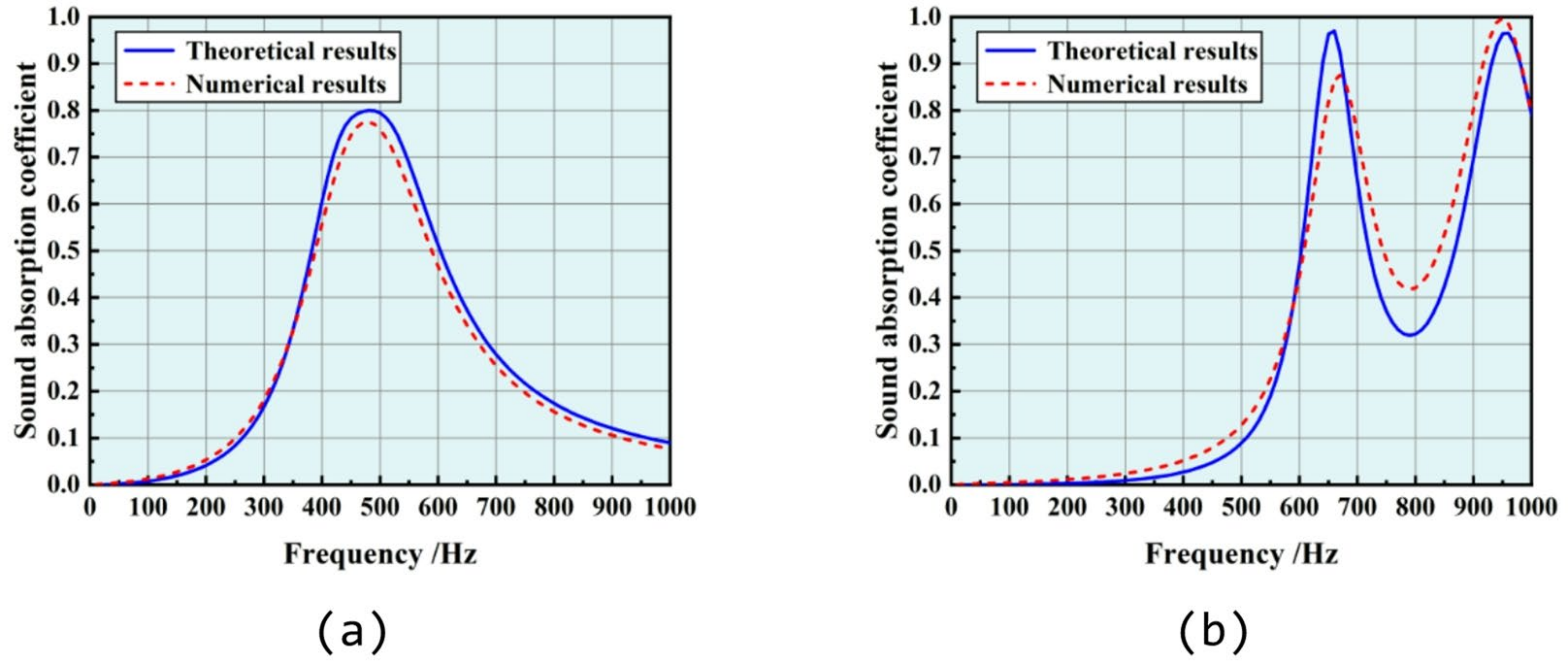
Comparison between theoretical (in blue) and numerical (in red) results in SAC evaluation: (**a**) case 1, (**b**) case 2. Geometric parameters of the two samples are listed in Table 1.

Since the theoretical model has been validated by both experimental and numerical results in our previous study^48–50^, it is employed to construct a dataset for the micro-perforated N–H cored sandwich metamaterial. Figures 2 present the theoretical results along with numerical validation of the structural SAC within 50 ~ 1000 Hz for two cases, with their parameters detailed in Table 1. In the dataset, the input data comprises the abovementioned structural parameters of the micro-perforated N–H cored sandwich metamaterial, while the targeted data consists of the SAC that is discretized into 20 sampling points with a step size of 50 H within 50 ~ 1000 Hz. The structural parameters are randomly generated within the selected data range in Table 2, and corresponding SAC is then calculated by the abovementioned theoretical model. 22,000 samples are generated and then partitioned into training, validation, and test datasets in a ratio of 9:1:1. The training dataset is used to train the model, through which the model learns the mapping relationship between inputs and outputs. The validation dataset is used to evaluate the model’s performance during the training process. The test dataset is used for the final evaluation of the model’s generalization ability. The test dataset consists of data that the model has not seen before, therefore it is used to assess the model’s actual application effectiveness. It has been verified that there is no data overlap across three datasets.

**Table 1:** The structural parameters of micro-perforated N–H cored sandwich metamaterial (mm).

	*a*	*b*	${d}_{f}$	${d}_{h}$	${d}_{c}$	*t*	*h*
Case 1	39.2	6	0.6	1.4	1.1	1.8	30
Case 2	47	6.7	1.5	1.9	1.4	2.9	30

**Table 2:** Ranges for structural parameters in dataset (mm).

	*a*	*b*	${d}_{f}$	${d}_{h}$	${d}_{c}$	*t*	*h*
Range	[30,50]	[5,8]	[0.5, 2]	[0.5, 2]	[0.5, 2]	[1,3]	30

The methodology for LLMs-based data-driven design for sound absorption performance of the micro-perforated N–H cored sandwich metamaterial is presented in this section. The agent interaction strategy is executed via dialogue with ChatGPT o4-mini-high, whereas the LLM fine-tuning strategy is implemented using Deepseek-R1-1.5b. For comparative analysis, a conventional ML model is developed based on a residual neural network (ResNet). The specific methodologies for agent interaction and LLM fine-tuning are elaborated in Sects. “Agent interaction strategy” and “LLM fine-tuning strategy”, respectively. The construction of ResNet architecture is detailed in the Sect. “Conventional ML model”.

### Agent interaction strategy

**Fig. 3:**
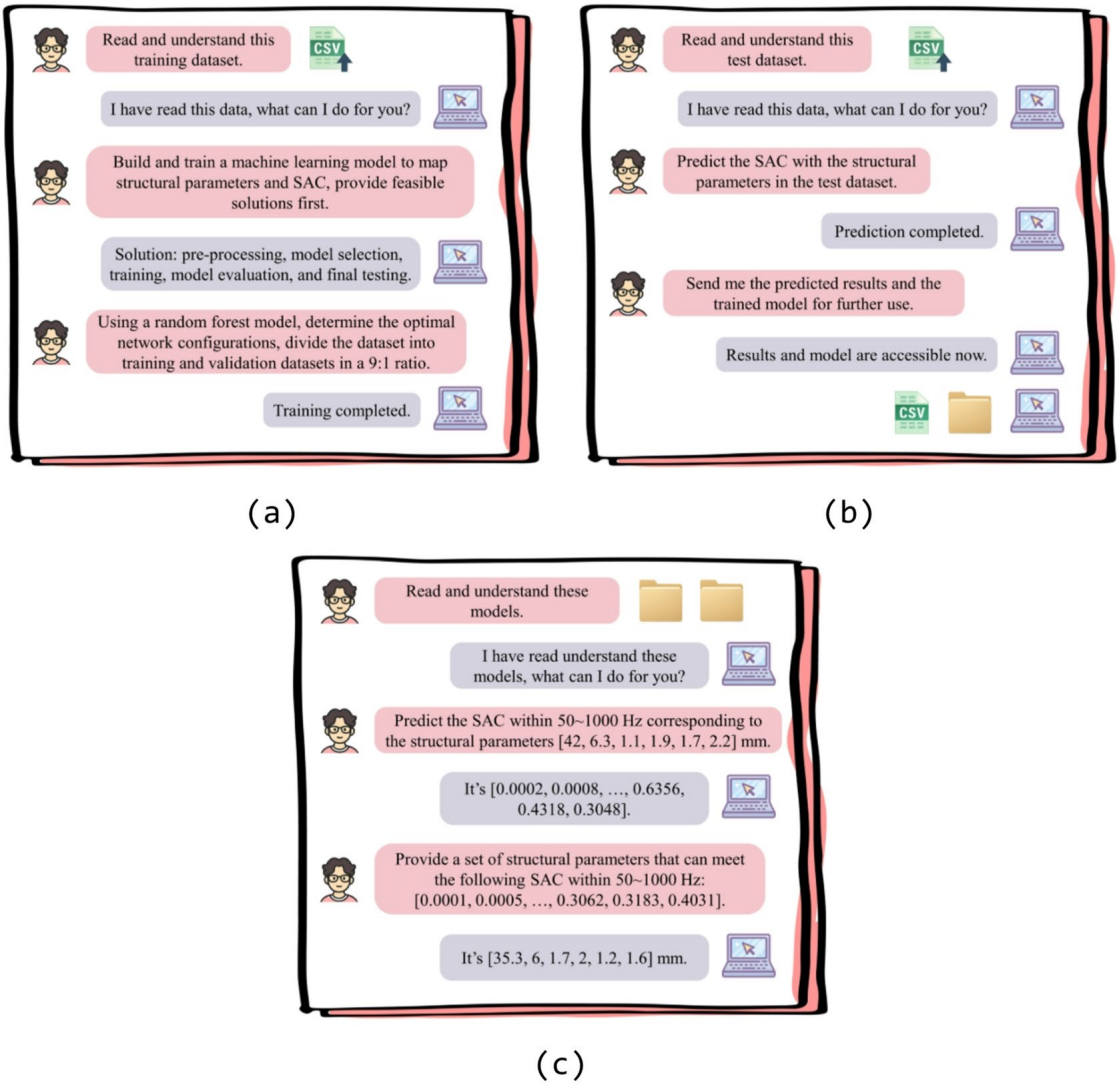
Key instructions for agent interaction strategy using ChatGPT o4-mini-high: (a) training process, (**b**) testing process and (**c**) further use.

Agents are endowed with perceptual capabilities, autonomy, and goal-directed behavior. Consequently, the agent interaction strategy enables the agent to discern designers’ interaction requirements through natural language in data-driven design tasks, make autonomous decisions, and execute them via logical reasoning, ultimately providing feedback on design outcomes in natural language. In this subsection, ChatGPT o4-mini-high is utilized as an AI agent for the agent interaction strategy owing to its superior abilities in coding and reasoning. Tasks such as data analysis, solution formulation, and model training are all carried out through dialogue commands with ChatGPT o4-mini-high. Importantly, this entire process is completed without necessitating any coding or knowledge of ML theory. Figure 3 delineates the pivotal instructions for executing the agent interaction strategy using ChatGPT o4-mini-high. The entire conversation can be segmented into three distinct parts as follows:

#### Training process

Using SAC forward prediction (i.e. prediction of SAC based on given structural parameters) as a case study, the training process (see in Fig. 3a) begins with uploading the training dataset to the dialogue interface. Initially, a confirmation command is issued to verify that ChatGPT has correctly received and interpreted the dataset. Upon receiving confirmation, ChatGPT is directed to establish a mapping from structural parameters to SAC and to propose initial feasible solutions. After reviewing the proposed plans, a subsequent instruction is given to instruct ChatGPT to partition the training dataset according to the specified ratio mentioned above and to train the model using the most precise methodology. Once feedback is obtained, the training process is concluded. Given the constraints of memory and computational power, ChatGPT is limited to training simpler ML models such as random forest and linear regression. Consequently, a random forest model is utilized in this study for the agent interaction strategy.

#### Testing process

In a similar manner, the testing process (see in Fig. 3b) requires uploading the test dataset and receiving confirmation of its receipt. Once confirmed, an instruction is issued to ChatGPT to predict the SAC for each sample in the test dataset. The predicted results and the trained model are then returned in a file format. Upon receiving ChatGPT’s response, these predicted results and the trained model are downloaded for further analysis and use.

#### Further use

Due to ChatGPT’s occasional forgetfulness, the trained model must be re-uploaded to the conversation for subsequent use (see in Fig. 3c). For example, by uploading the trained forward prediction and inverse design (i.e. identification of structural parameters according to given SAC values) models to the dialogue, ChatGPT can be prompted to perform SAC prediction and on-demand design tasks as needed.

### LLM fine-tuning strategy

Although LLMs like ChatGPT-4 are inherently adaptable and capable of handling a wide range of language text tasks, most general LLMs have not been exposed to sufficient domain-specific data, leading to difficulties in understanding and tackling domain-specific problems. LLM fine-tuning is the process of taking a pre-trained LLM model and further training it on a specific dataset to adapt it to a particular task or domain. This involves adjusting the model’s parameters based on the new data to improve its performance and relevance for the targeted application. The fine-tuning process leverages the general language understanding capabilities of the pre-trained model while tailoring it to handle specific types of data, tasks, or domain-specific knowledge more effectively.

From the perspective of parameter scalability, the fine-tuning of LLMs diverges into two principal methodological pathways: one employs full-parameter optimization, formally termed Full Fine-Tuning (FFT), whereas the alternative adopts a selective updating strategy that targets only a subset of parameters, a technique characterized as Parameter-Efficient Fine-Tuning (PEFT). FFT are computationally prohibitive due to the massive parameter counts (billions to trillions) of modern LLMs, require substantial storage for each task-specific model variant, and risk catastrophic forgetting of the model’s original capabilities. To mitigate the limitations mentioned above, we employ PEFT, which updates only a small—or even minimal—subset of model parameters. This approach significantly reduces computational and storage costs, better preserves the general knowledge acquired during pretraining, and effectively mitigates the issue of catastrophic forgetting.

Low-Rank Adaptation (LoRA) represents a typical PEFT methodology, it operates under the constraint of low-rank matrix decomposition to facilitate efficient model adaptation^51,52^. In the fine-tuning process, let $\text{h}=\text{Wx}$ be the original forward pass in a given layer, where $\text{x}\in {\mathbb{R}}^{k\times 1}$ is the input and $\text{h}\in {\mathbb{R}}^{d\times 1}$ is the output. The objective of fine-tuning is to find an optimal update matrix $\Delta W$ such that the new output $h^{\prime }$ is:

8 $$\begin{array}{*{20}c} {h^{\prime } = \left( {W + \Delta W} \right)x} \\ \end{array}$$

FFT learns the full matrix $\Delta W \in {\mathbb{R}}^{d \times k}$ directly, which is computationally expensive as it requires updating and storing $d \times k$ parameters. LoRA circumvents this by imposing a low-rank constraint on $\Delta W$. Specifically, it decomposes the update matrix into the product of two smaller matrices, ${\mathbf{A}} \in {\mathbb{R}}^{d \times r}$ and ${\mathbf{B}} \in {\mathbb{R}}^{r \times k}$, where the rank $r$ is chosen such that $r \ll \min \left( {d,k} \right)$.

9 $$\begin{array}{*{20}c} {\Delta W = BA} \\ \end{array}$$

Therefore, the adapted forward pass in LoRA is formulated as:

10 $$\begin{array}{*{20}c} {h^{\prime } = Wx + \Delta Wx = Wx + BAx} \\ \end{array}$$

where $W \in {\mathbb{R}}^{d \times k}$ is the pre-trained weight matrix, which is frozen during training. The only trainable parameters are the matrices ${\mathbf{A}}$ and ${\mathbf{B}}$. This architectural constraint reduces the number of trainable parameters from $d \times k$ to $r\left( {d + k} \right)$. The parameter efficiency inherent to LoRA arises directly from its low-rank factorization, with the reduction in trainable parameters quantified by the ratio:

11 $$\begin{array}{*{20}c} {\frac{{r\left( {d + k} \right)}}{d \times k} = r\left( {\frac{1}{k} + \frac{1}{d}} \right)} \\ \end{array}$$

Given the condition that $r \ll \min \left( {d,k} \right)$, this ratio remains substantially small. A pivotal design consideration in LoRA involves the initialization of the factor matrices ${\text{A}}$ and ${\text{B}}$. Conventionally, ${\text{A}}$ is initialized using a random Gaussian distribution, $A \sim {\mathcal{N}}\left( {0,\sigma^{2} } \right)$, whereas ${\text{B}}$ is initialized as a null matrix, ${\text{B}} = 0$. This initialization strategy ensures that the combined update matrix $\Delta W = BA$ is zero at the beginning of training.

**Fig. 4:**
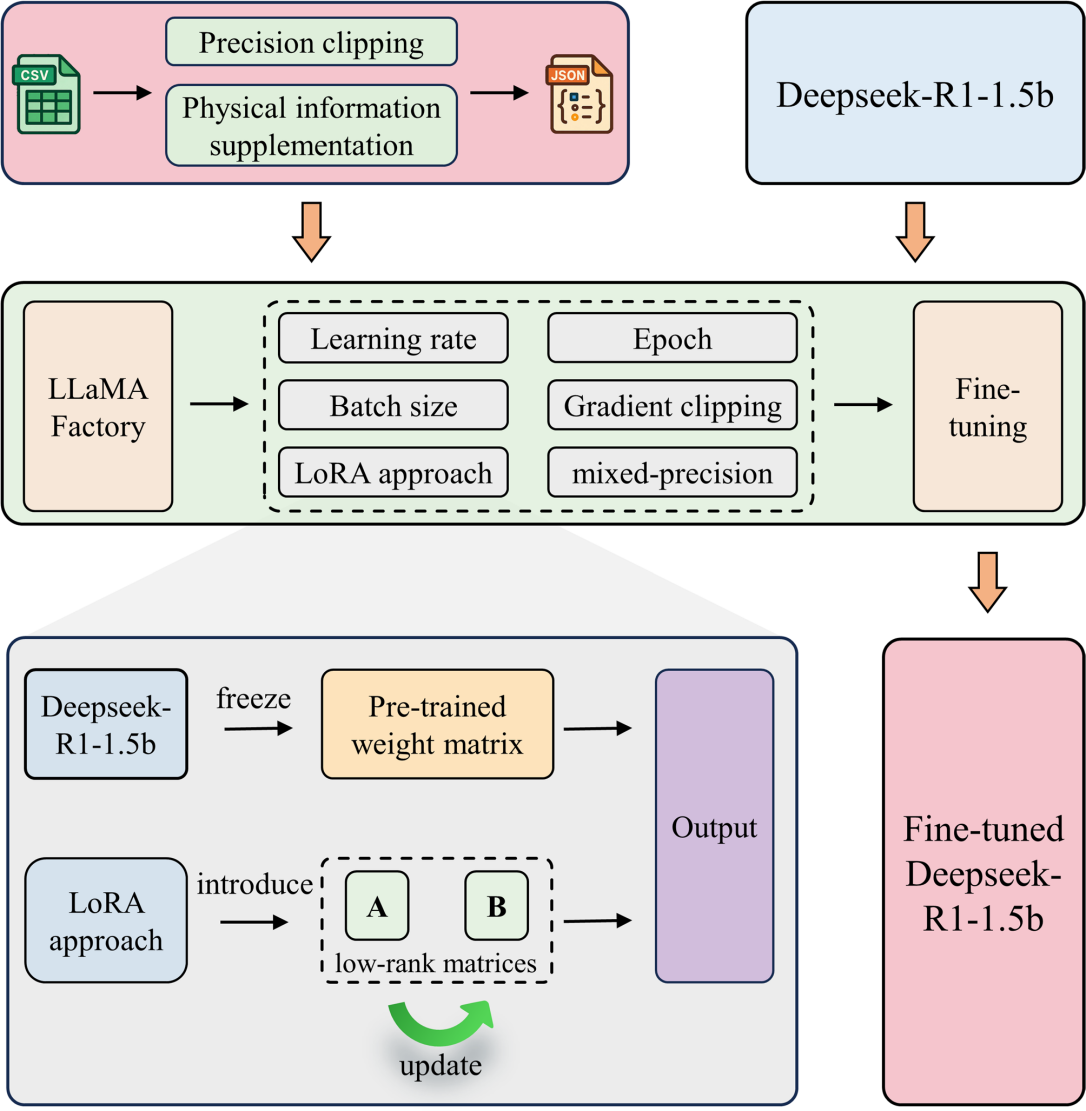
Overview of LLM fine-tuning strategy.

Figure 4 illustrates the process of LLMs fine-tuning strategy, which can be can be summarized in three distinct phrases (see Fig. 4) as follows:

#### Data preprocessing

It is essential to acknowledge that the original design intent of LLMs is indeed to process natural language rather than perform numerical calculation. Consequently, LLMs are primarily trained on vast amounts of natural language text, where numbers typically appear in specific contexts. This leads to a lack of extensive and diverse training on pure numerical data, thereby impacting their ability to directly understand numbers. For instance, numerical values such as 99 and 99.00 are often processed as semantically distinct entities by LLMs, a discrepancy that undermines their performance in data prediction tasks. To enhance the performance of LLMs in data prediction tasks, we implemented four specialized operations on the dataset. Initially, structural parameters and SAC values within the dataset were rounded to four decimal places. This precision level was selected to balance numerical accuracy, which can be compromised by excessive rounding, against tokenization efficiency, as overly precise values can result in fragmented tokenization and thereby degrade model training performance. Subsequently, a dictionary was constructed to map key structural parameters to their corresponding physical interpretations in natural language. This approach enhances the learning process of LLMs by providing contextual prompts, which improves semantic comprehension of the data and leads to superior training outcomes. Similarly, another dictionary is constructed to store the SAC values and their associated frequencies. Finally, all the information is converted into JavaScript Object Notation (JSON) format for the subsequent LLM fine-tuning. JSON, as a syntax for storing and exchanging textual information, provides a highly standardized and hierarchical data structure that facilitates efficient parsing within LLMs.

#### Fine-tuning

The fine-tuning of Deepseek-R1-1.5b is carried out using LLaMA-Factory which is a powerful and efficient framework for fine-tuning LLMs. LLaMA-Factory enables practitioners from non-AI fields to easily fine-tune LLMs through an intuitive user interface and a comprehensive set of features, thereby eliminating the need for manual coding. During the fine-tuning process, the initial learning rate is set to 0.0005 and follows a cosine annealing decay schedule. Employing a mixed-precision training scheme that integrates single-precision (float32) and half-precision (float16) arithmetic combines the memory efficiency and accelerated computation of half-precision operations with enhanced numerical stability, thereby mitigating the risk of overflow and loss of precision associated with exclusive use of lower-precision formats. To improve the stability of fine-tuning, a gradient clipping strategy with a threshold of 1.0 is employed to prevent gradient explosion. Additionally, the batch size is set to 3, and the fine-tuning process for Deepseek-R1-1.5b concludes after 3 epochs.

#### Tasks performing

Once the fine-tuning is completed, designers can instruct the fine-tuned Deepseek-R1-1.5b model to perform forward prediction and inverse design tasks. In addition, it’s important to note that when executing design tasks, the natural language and data format used in the designer’s instructions need align with the data preprocessing steps.

### Conventional ML model

The conventional ML model ResNet is also developed for comparative analysis with the abovementioned LLMs-based data-driven methods. ResNet incorporates two distinctive architectural features: shortcut connection and residual learning. Shortcut connection helps mitigate the vanishing gradient problem that tends to arise during the training of conventional ML models with deep architectures, while residual learning enhances the feature extraction capabilities of ResNet, allowing it to capture complex patterns more effectively^53,54^. Additionally, the unique architecture of ResNet contributes to improved training efficiency and computational performance. Owing to superior performance and reduced overhead in data-driven design applications, ResNet is employed in this subsection to train the SAC data.

**Fig. 5:**
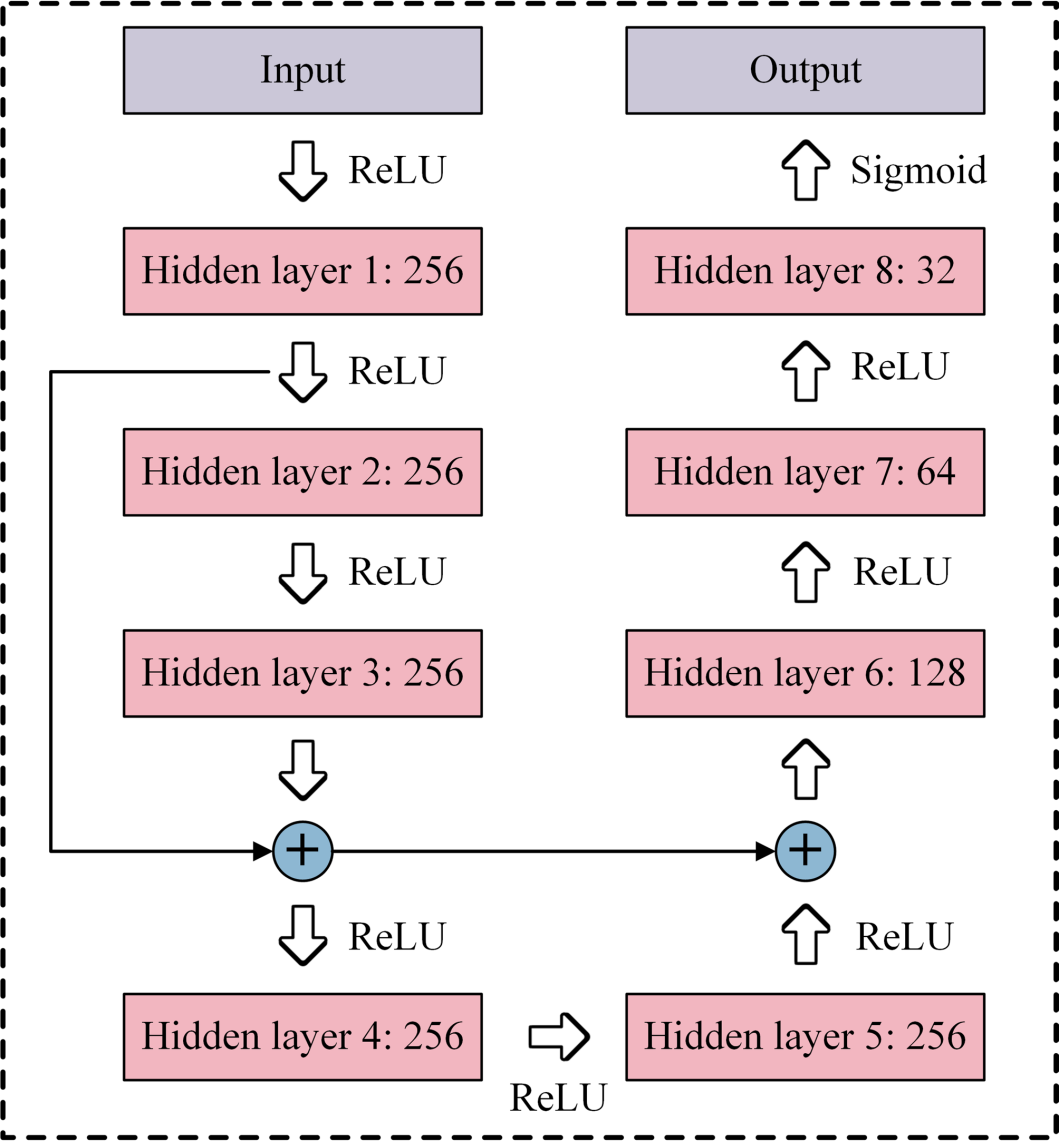
Schematic diagram of the ResNet architecture.

Figure 5 illustrates the ResNet architecture, which consists of an input layer, an output layer, eight hidden layers, and two residual connections. ReLU and Sigmoid are adopted as the activation functions in order to enhance the non-linear fitting ability of the network. During the training process, the batch size, learning rate and number of epochs are set to 512, 0.0005 and 300, respectively. In the training process for SAC forward prediction, structural parameters serve as inputs, while the SAC acts as the output. Conversely, the roles are reversed in the training for SAC inverse design.

## Results and discussion

### SAC forward prediction

Four samples from test dataset are selected to demonstrate the results of agent interaction and LLM fine-tuning strategy. The results of ResNet are also presented for better comparison. The structural parameters of four samples are listed in Table 3.

**Table 3:** Structural parameters of test samples (mm).

	*a*	*b*	${d}_{f}$	${d}_{h}$	${d}_{c}$	*t*	*h*
Sample 1	49.6	5.7	1.2	1.2	0.6	2.6	30
Sample 2	47.6	7.9	1.6	1.2	1.1	1.1	30
Sample 3	32	7.5	1.4	2.0	0.8	1.2	30
Sample 4	45	6.6	0.6	1.0	1.5	2.1	30

**Fig. 6:**
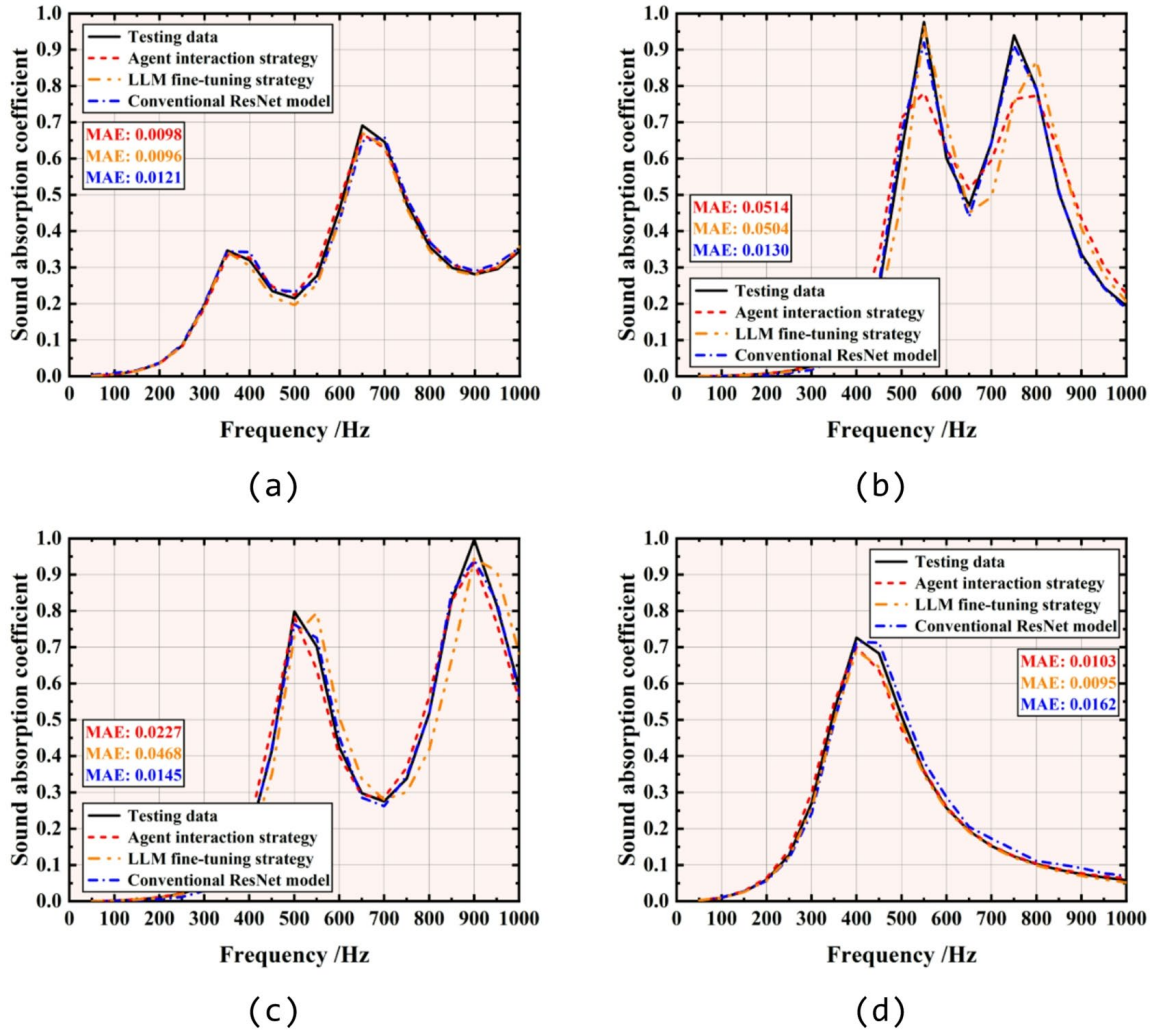
Comparison between the predicted SAC by agent interaction strategy (in red), LLM fine-tuning strategy (in orange) and conventional ResNet model (in blue) with the testing SAC (in black) from the test dataset: (**a**) sample 1, (**b**) sample 2, (**c**) sample 3 and (d) sample 4. Structural parameters of test samples are listed in Table 3.

**Fig. 7:**
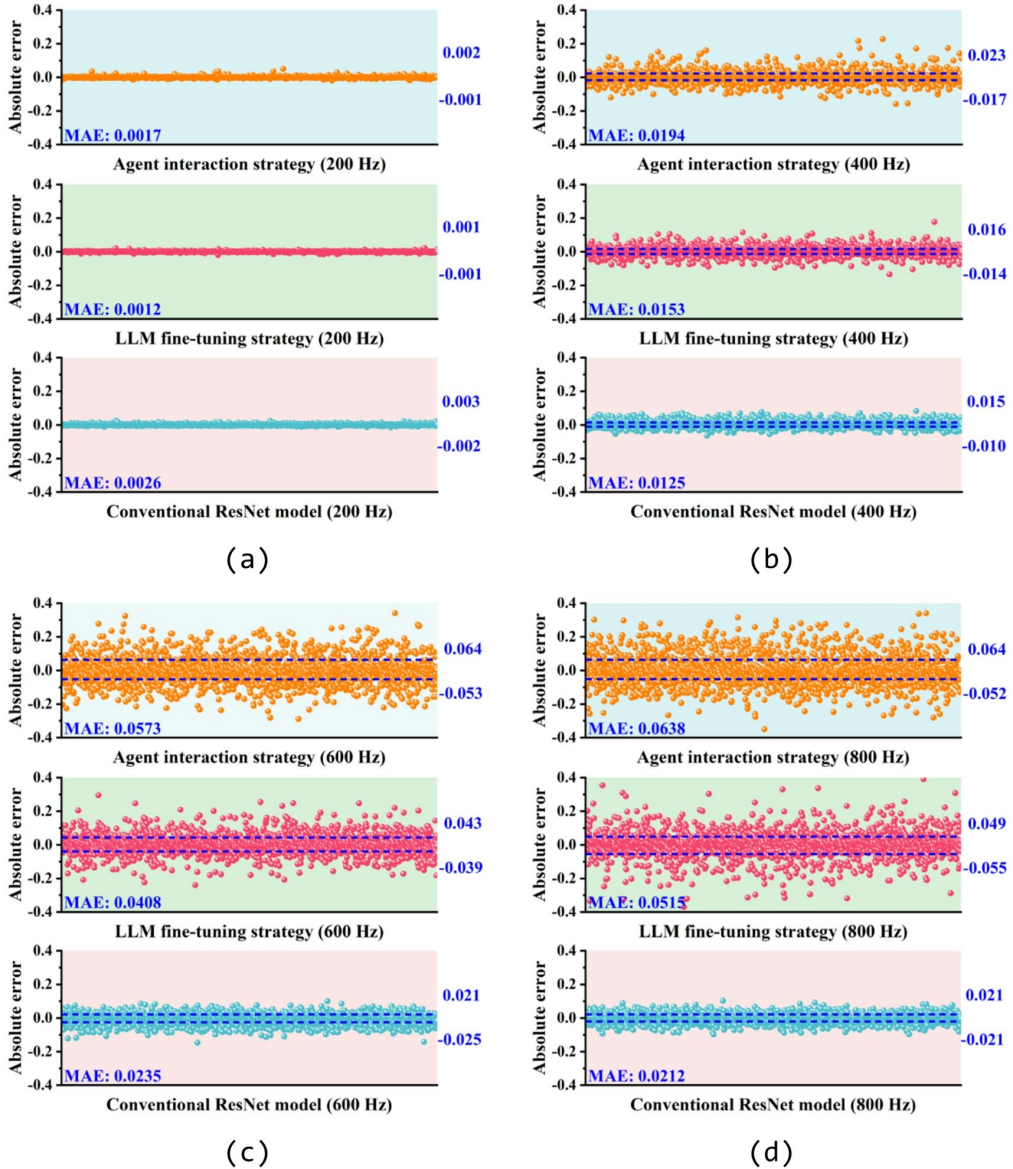
Error analysis of the predicted SAC by agent interaction strategy (in orange), LLM fine-tuning strategy (in red) and conventional ResNet model (in blue) compared with testing SAC from the test dataset at (**a**) 200 Hz, (**b**) 400 Hz, (**c**) 600 Hz and (d) 800 Hz. All the 2000 test samples in the test dataset are involved in the analysis.

The SAC forward prediction results by agent interaction strategy, LLM fine-tuning strategy, and conventional ResNet model are compared in Fig. 6. It’s evident that all three methods exhibit similar peak frequencies and curve trends compared with the SAC values from the test dataset. However, as shown in Fig. 6b, agent interaction strategy tends to provide a relatively conservative prediction result near the peak frequency, resulting in lower SAC peaks. Additionally, it can be observed in Fig. 6b, c that the results given by LLM fine-tuning strategy exhibit a peak frequency error of around 50 Hz compared to testing SAC. Based on the mean absolute error (MAE) between the predicted and test values of each method shown in Fig. 6, it is evident that the conventional ML model demonstrates a better accuracy and stability in SAC prediction for all four samples. To further assess the generalization capability of the agent interaction and LLM fine-tuning strategy, Fig. 7 present the absolute errors between the predicted results and testing SAC values of 2000 samples in the test dataset at 200 Hz, 400 Hz, 600 Hz and 800 Hz. Results indicate that at lower frequencies, both the agent interaction and LLM fine-tuning strategy, along with conventional ML model, exhibit low errors across 2000 testing samples. However, the absolute errors of all three methods increase as the frequency rises. In contrast, based on the MAE shown in Fig. 7, the conventional ML model demonstrates the highest stability in SAC prediction at higher frequencies, with a maximum MAE not exceeding 0.0235. This is followed by the LLM fine-tuning and agent interaction strategies, with maximum MAEs of 0.0515 and 0.0638, respectively.

The discrepancies can be attributed to follow reasons: for the agent interaction strategy, limitations in memory and computing power restrict it to using simpler ML models such as random forest and linear regression. As a result, agent interaction strategy struggles to achieve the same accuracy in predicting high-frequency SAC with larger fluctuations as it does in predicting low-frequency SAC with smaller fluctuations. For LLM fine-tuning strategy, the retraining outcome of LLM depend on the volume of data it employs during the training. Additionally, the training strategy and hyperparameters employed during fine-tuning inevitably contribute to the errors.

### SAC inverse design

Figure 9 illustrate the inverse design outcomes achieved using the agent interaction, LLM fine-tuning strategy and the conventional ResNet model.

**Fig. 8:**
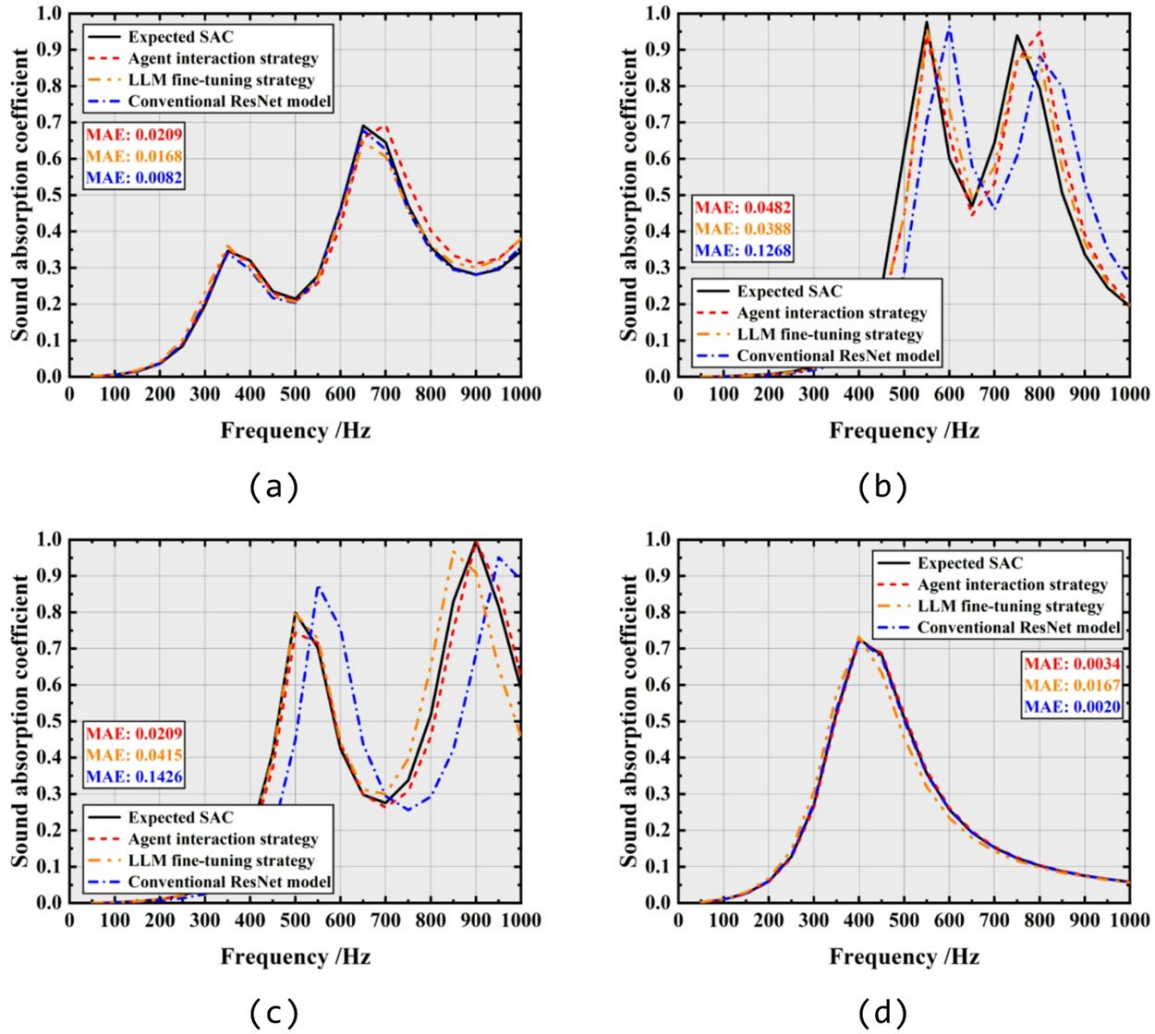
Designed structural parameters of (**a**) target 1, (**b**) target 2, (**c**) target 3 and (**d**) target 4 given by agent interaction strategy, LLM fine-tuning strategy and the conventional ResNet model.

**Fig. 9:**
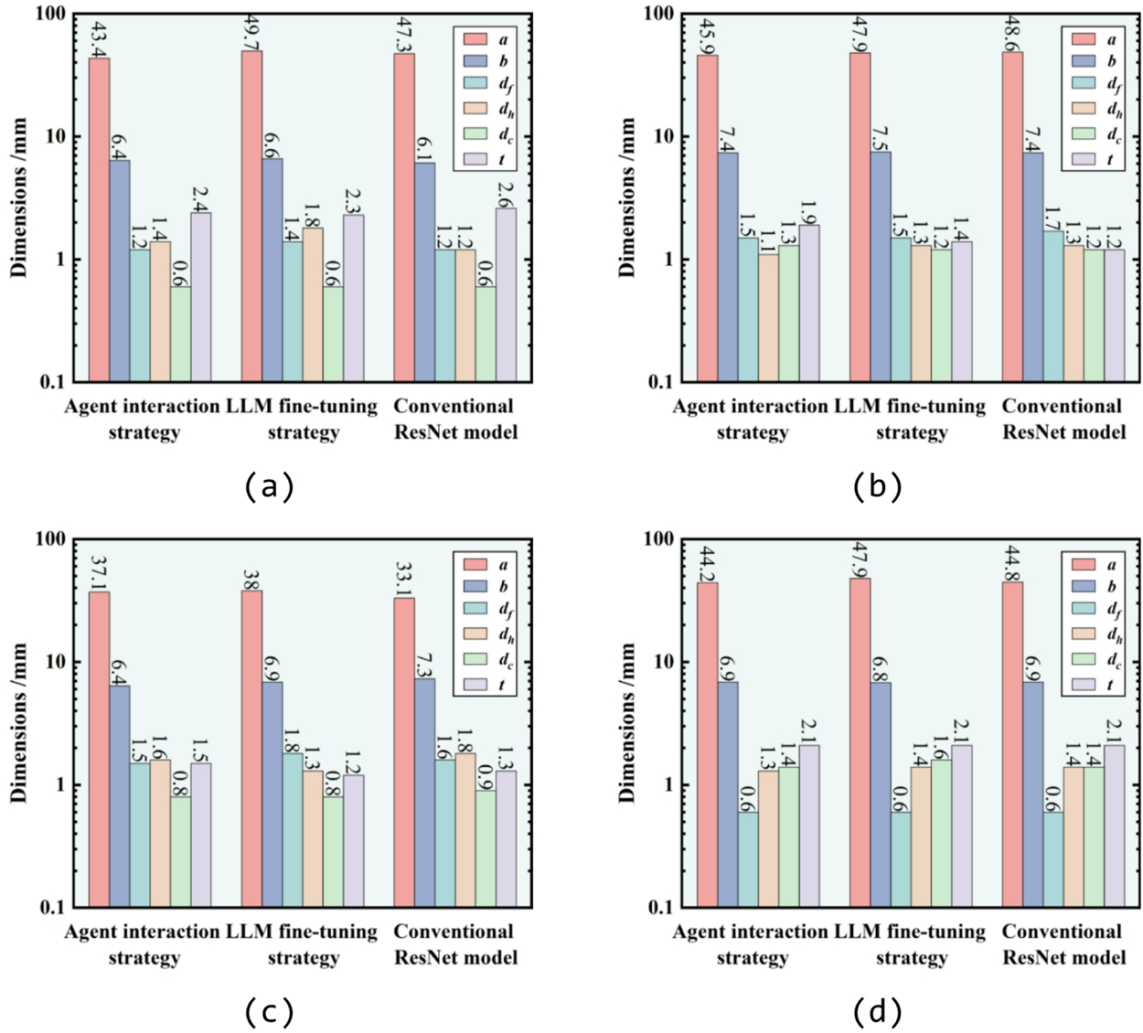
SAC comparison between the designed samples by agent interaction strategy (in red), LLM fine-tuning strategy (in orange) and conventional ResNet model (in blue) with the expected SAC (in black): (**a**) target 1, (**b**) target 2, (**c**) target 3 and (**d**) target 4. Structural parameters corresponding to expected SAC are listed in Table 3.

**Fig. 10:**
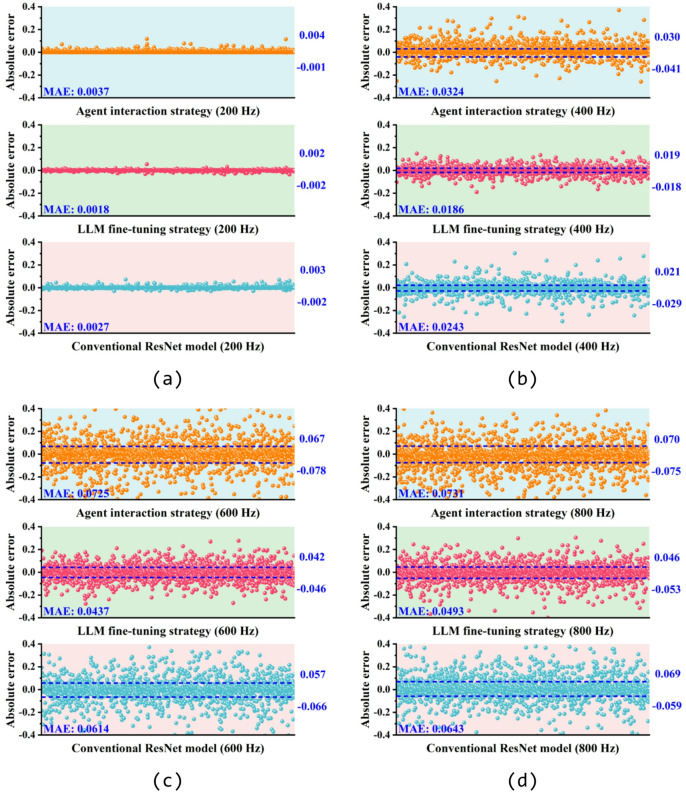
SAC error analysis of the designed samples by agent interaction strategy (in orange), LLM fine-tuning strategy (in red) and conventional ResNet model (in blue) compared with expected SAC at (**a**) 200 Hz, (**b**) 400 Hz, (**c**) 600 Hz and (**d**) 800 Hz. All the 2000 test samples in the test dataset are involved in the analysis.

For the inverse design, the SAC values from four samples in the test dataset are employed as the target specifications. The corresponding structural parameters from the test dataset are the same as that listed in Table 3. The design results provided by the agent interaction, LLM fine-tuning strategy and conventional ResNet model are presented in Fig. 8. It is observed that, the three methods yield similar design parameters for each target SAC. Detailed structural parameters generated by the three methods are listed in Tables 4, 5 and 6, respectively. To better compare the design results of the three methods, the four sets of structural parameters are input into the theoretical model mentioned above^48–50^ to calculate SAC. As can be seen in Fig. 9d, all three methods yield designs that nearly achieve the target SAC for inverse design with a single-peak characteristic. However, as illustrated in Fig. 9b and c, for inverse design with expected SAC characterized by multi-peaks, although results from the conventional ML model present similar curve trends, deviations near the peak frequencies are observed. In contrast, the results from agent interaction and LLM fine-tuning strategy exhibit smaller error in peak frequency across all four designs. The MAE presented in Fig. 9 also indicates the instability of the conventional ML model in SAC inverse design, whereas agent interaction strategy exhibits improved stability and accuracy. Figure 10 present the generalization ability of the three methods for SAC inverse design. As observed, the absolute errors between designed SAC and target SAC by three methods increase as frequency rises. However, different from that of forward design, the accuracy of the results by agent interaction strategy is nearly identical to that of ResNet, while the LLM fine-tuning strategy demonstrates the best accuracy.

**Table 4:** Designed structural parameters by agent interaction strategy (mm).

	*a*	*b*	${d}_{f}$	${d}_{h}$	${d}_{c}$	*t*	*h*
Design 1	43.4	6.4	1.2	1.4	0.6	2.4	30
Design 2	45.9	7.4	1.5	1.1	1.3	1.9	30
Design 3	37.1	6.4	1.5	1.6	0.8	1.5	30
Design 4	44.2	6.9	0.6	1.3	1.4	2.1	30

**Table 5:** Designed structural parameters given by fine-tuning strategy (mm).

	*a*	*b*	${d}_{f}$	${d}_{h}$	${d}_{c}$	*t*	*h*
Design 1	49.7	6.6	1.4	1.8	0.6	2.3	30
Design 2	47.9	7.5	1.5	1.3	1.2	1.4	30
Design 3	38	6.9	1.8	1.3	0.8	1.2	30
Design 4	47.9	6.8	0.6	1.4	1.6	2.1	30

**Table 6:** Designed structural parameters by conventional ResNet model (mm).

	*a*	*b*	${d}_{f}$	${d}_{h}$	${d}_{c}$	*t*	*h*
Design 1	47.3	6.1	1.2	1.2	0.6	2.6	30
Design 2	48.6	7.4	1.7	1.3	1.2	1.2	30
Design 3	33.1	7.3	1.6	1.8	0.9	1.3	30
Design 4	44.8	6.9	0.6	1.4	1.4	2.1	30

In addition to the discussion in the previous subsection (see Sect. “SAC forward prediction”), the discrepancy can also be attributed to the fact that the inverse design of SAC is a multi-solution problem, and the non-uniqueness of the mapping from SAC to structural parameters makes the inverse task more complex, resulting in errors greater than those observed in forward prediction for all three methods. Overall, as shown in Fig. 10, the MAE by agent interaction strategy does not exceed 0.0731, while LLM fine-tuning strategy exhibits a MAE of no more than 0.0493, which is better than 0.0643 by conventional ML model. Compared to agent interaction strategy, the LLM fine-tuning strategy exhibits higher accuracy in both forward and inverse design.

### Further discussion

**Fig. 11:**
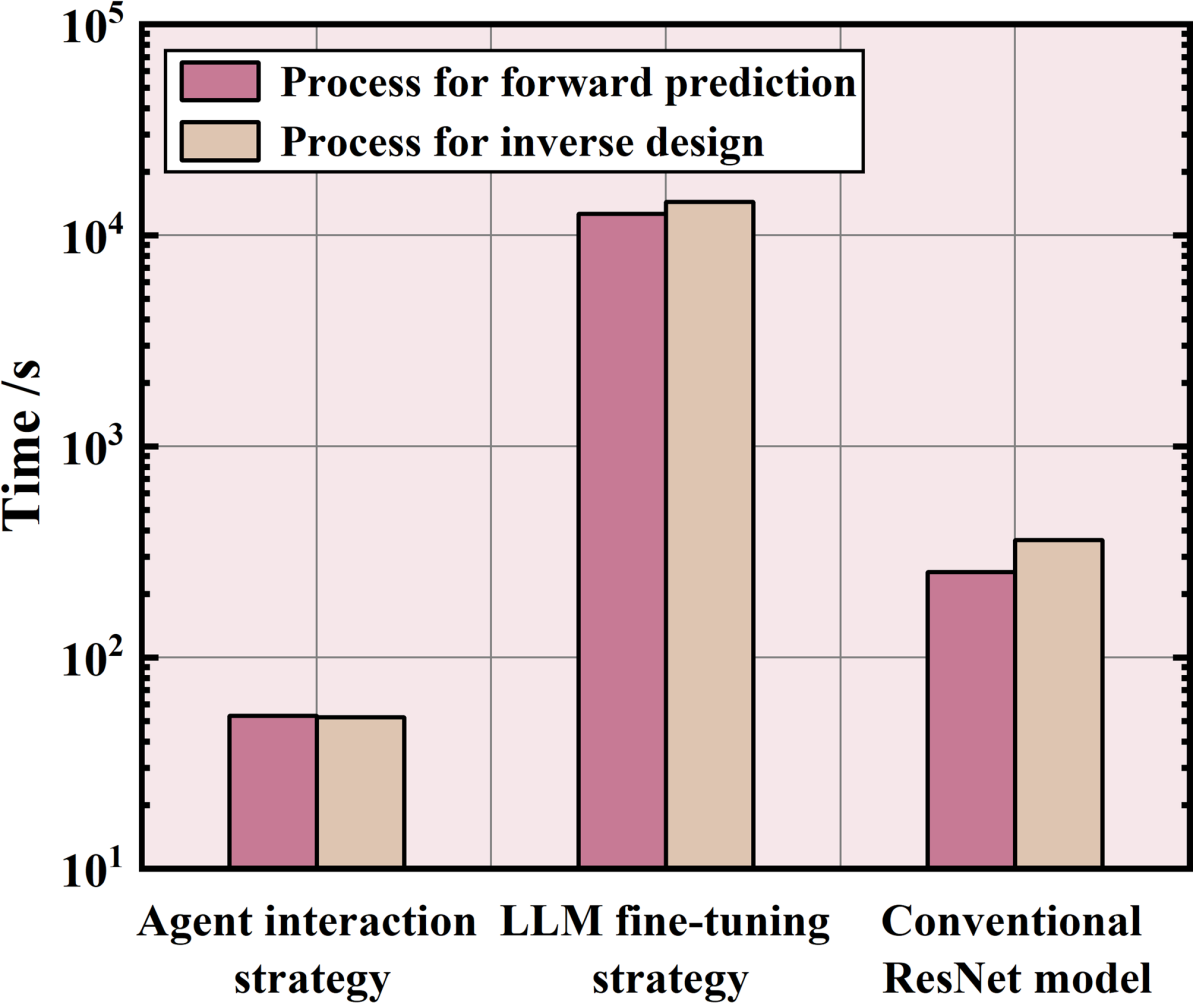
Time consumption of agent interaction strategy, LLM fine-tuning and conventional ResNet model for SAC forward prediction and inverse design process (GPU: Nvidia GeForce RTX 4090 D).

This subsection provides a comprehensive discussion based on qualitative analysis to evaluate the characteristics of the agent interaction strategy, LLM fine-tuning strategy, and conventional ML methods in SAC forward prediction and inverse design. Five criteria are considered: data processing capacity, simplicity, accuracy, adaptability, and efficiency. The three methods are then ranked according to each of these evaluation metrics. Firstly, as a LLM with billions of parameters, the fine-tuned Deepseek demonstrates superior data processing capabilities compared to both the agent interaction strategy and the conventional ResNet model. This enables it to learn from large-scale datasets and diverse knowledge. In contrast, the agent interaction strategy is limited to handling smaller-scale datasets because it is executed within a chat interface. Secondly, the agent interaction strategy relies entirely on dialogue, making it the simplest data-driven approach. In contrast, conventional ResNet model tends to be most challenging to implement because building ResNet model requires designers to be proficient in both coding and ML theory. Accuracy ranking is determined according to the results presented in Sects. “SAC forward prediction” and “SAC inverse design”. Considering adaptability, the agent interaction strategy does not require high-performance CPUs or GPUs hardware, whereas the other two strategies necessitate robust hardware resources. Among them, the LLM fine-tuning strategy presents the most stringent GPU requirements due to the complex model structure and parameters. Finally, the efficiency ranking is determined based on the time required for each strategy to complete the design task. Figure 11 illustrates the time consumption of the three methods for SAC forward prediction and inverse design process including data process, model training and testing. It is evident that the agent interaction strategy can complete data processing, model training and other work in under 1 min for both SAC forward prediction and inverse design. However, the conventional ResNet model requires about 5 min to complete a training, and it takes three to four hours to complete a fine-tuning of DeepSeek. The GPU used in this study is Nvidia GeForce RTX 4090 D.

**Fig. 12:**
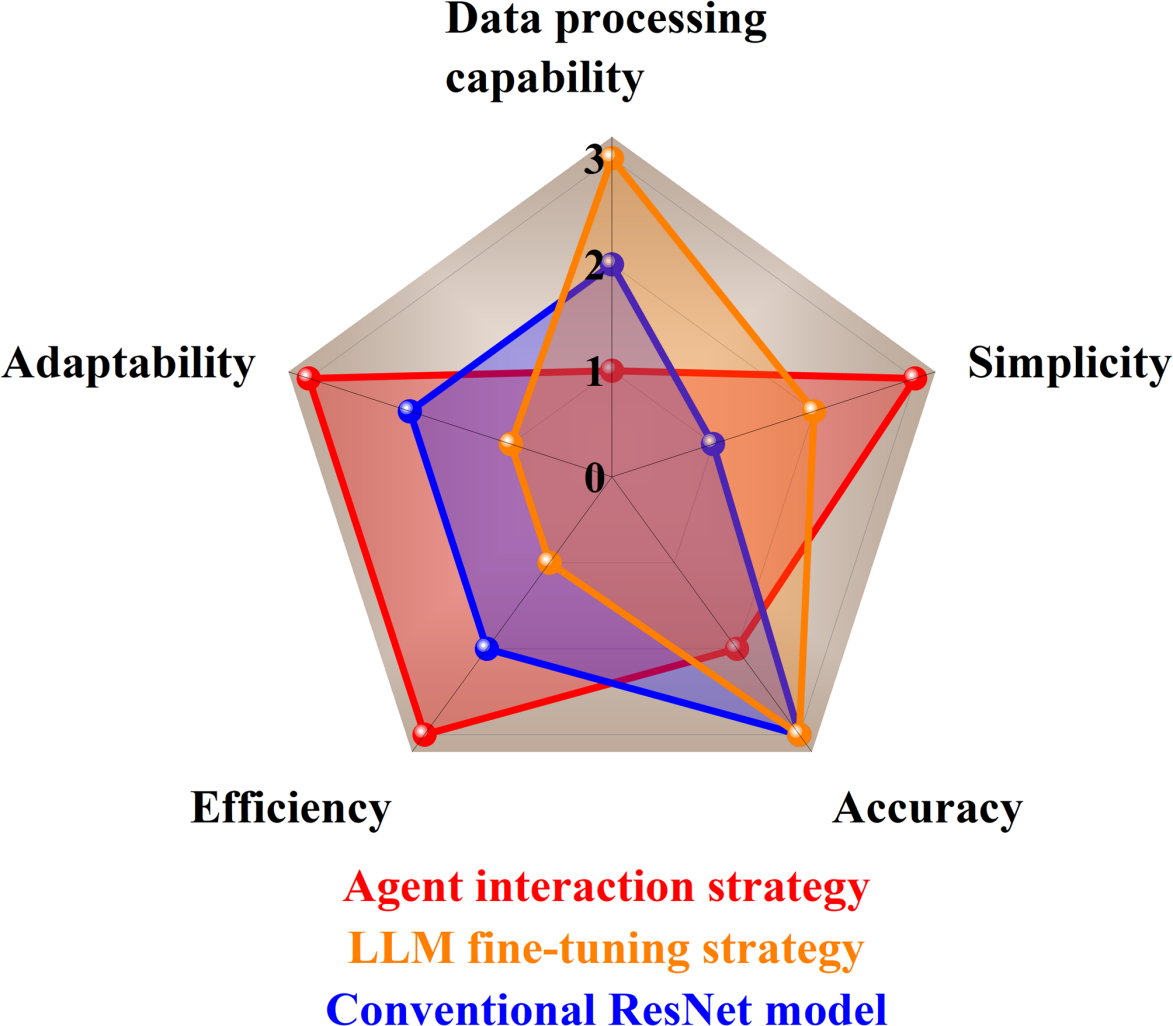
A radar chart for evaluating the comprehensive characteristics of agent interaction strategy (in red), LLM fine-tuning (in orange) and conventional ResNet model (in blue).

A radar chart shown in Fig. 12 displays the comprehensive evaluation of the three data-driven design methods of sound absorption performance of AMM. The results show that the agent interaction strategy excels in simplicity, adaptability, and efficiency, while the LLM fine-tuning strategy stands out for its accuracy and data processing capability. In summary, agent interaction strategy provides a new paradigm for lightweight data-driven tasks, while LLM fine-tuning strategy is better suited for large-scale data-driven tasks. Both methods can be performed without requirements of professional knowledge of ML.

## Conclusions

This paper presented an investigation on data-driven design of sound absorption performance of AMMs based on LLMs. The agent interaction and LLM fine-tuning strategies were proposed based on ChatGPT and Deepseek respectively in this study. The agent interaction strategy, developed with ChatGPT o4-mini-high, demonstrated that designers can establish correlations between structural and sound absorption performance of AMMs via dialogue and instructions, facilitating both forward prediction and reverse design. Deepseek-R1-1.5b was trained using existing datasets of AMMs in LLM fine-tuning strategy. The fine-tuned Deepseek demonstrated accuracy comparable to conventional ResNet model in both forward prediction and inverse design of AMM sound absorption performance. Moreover, the LLM fine-tuning strategy makes it possible for fine-tuned Deepseek to evolve into an AMM-specific model through on-going knowledge integration. A qualitative analysis focusing on data processing capability, simplicity, accuracy, efficiency, and adaptability was conducted to evaluate the comprehensive characteristics of the two strategies. The results indicated that the agent interaction strategy excels in efficiency, adaptability, and simplicity while maintaining accuracy. In contrast, the LLM fine-tuning strategy demonstrates superior accuracy and data processing capability. In conclusion, the two strategies highlight the simplicity and progressive nature of LLM-based data-driven design. With the assistance of LLM, data-driven design is no longer only accessible to designers with ML expertise but to all designers. The strategies outlined in this study are expected to push data-driven design towards enhanced intelligence and user-friendliness. It is noteworthy that while LLM-based strategies have proven effective and user-friendly for the design of acoustic metamaterials, they currently have certain limitations. For instance, since LLMs fundamentally learn and operate through natural language, any design strategy based on them requires translating data into this format first. This process poses a significant challenge when dealing with complex data forms like images and videos, as their detailed description in natural language is inherently difficult. In addition, compared to traditional small machine learning models, LLMs might not necessarily perform better in terms of accuracy, generalization, and complex mapping problem solving. The effectiveness of proposed methods in different domains requires further validation. Moreover, although this study demonstrates the feasibility of LLM-based approaches for designing acoustic metamaterials, several promising avenues for future exploration remain. A primary direction for future work is to apply these strategies to the design of other metamaterials, such as photonic crystals, thermal cloaks, or mechanical metamaterials. Once generalizability of the LLM-based approaches is validated, they can be established as universal tool for structural design. Furthermore, the proposed strategies would also incorporate more advanced LLM architectures or fine-tuning techniques with enhanced reasoning capabilities or be combined with traditional ML models to improve their design accuracy and tackle more complex problems. Moreover, the proposed methods could be combined with advanced manufacturing techniques, such as 3D printing, to form an automated design-to-fabrication pipeline, i.e., promising structures generated by the LLM are selected automatically and fabricated via 3D printing, their acoustic properties are then experimentally characterized. The results from experimental tests are fed back into the model’s knowledge base as new data points.

## Data Availability

The datasets used and/or analyzed during the current study available from the corresponding author on reasonable request.
